# Extracellular Vesicles from Ovarian Carcinoma Cells Display Specific Glycosignatures

**DOI:** 10.3390/biom5031741

**Published:** 2015-08-04

**Authors:** Joana Gomes, Patrícia Gomes-Alves, Sofia B. Carvalho, Cristina Peixoto, Paula M. Alves, Peter Altevogt, Julia Costa

**Affiliations:** 1 Instituto de Tecnologia Química e Biológica António Xavier, Universidade Nova de Lisboa, Av. da República, Oeiras 2780-157, Portugal; E-Mails: jgomes@itqb.unl.pt (J.G.); palves@itqb.unl.pt (P.G.-A.); sofiacarvalho@itqb.unl.pt (S.B.C.); peixoto@itqb.unl.pt (C.P.); marques@itqb.unl.pt (P.M.A.); 2 iBET, Instituto de Biologia Experimental e Tecnológica, Oeiras 2780-157, Portugal; 3 Skin Cancer Unit, German Cancer Research Center (DKFZ), Heidelberg 69120, Germany; E-Mail: p.altevogt@dkfz.de; 4 Department of Dermatology, Venereology and Allergology, University Medical Center Mannheim, Ruprecht-Karl University of Heidelberg, Mannheim 68135, Germany

**Keywords:** glycosylation, extracellular vesicles, ovarian cancer, galectin-3-binding protein, glycosignatures, kifunensine, biomarkers

## Abstract

Cells release vesicles to the extracellular environment with characteristic nucleic acid, protein, lipid, and glycan composition. Here we have isolated and characterized extracellular vesicles (EVs) and total cell membranes (MBs) from ovarian carcinoma OVMz cells. EVs were enriched in specific markers, including Tsg101, CD63, CD9, annexin-I, and MBs contained markers of cellular membrane compartments, including calnexin, GRASP65, GS28, LAMP-1, and L1CAM. The glycoprotein galectin-3 binding protein (LGALS3BP) was strongly enriched in EVs and it contained sialylated complex *N*-glycans. Lectin blotting with a panel of lectins showed that EVs had specific glycosignatures relative to MBs. Furthermore, the presence of glycoproteins bearing complex *N*-glycans with α2,3-linked sialic acid, fucose, bisecting-GlcNAc and LacdiNAc structures, and O-glycans with the T-antigen were detected. The inhibition of *N*-glycosylation processing from high mannose to complex glycans using kifunensine caused changes in the composition of EVs and induced a decrease of several glycoproteins. In conclusion, the results showed that glycosignatures of EVs were specific and altered glycosylation within the cell affected the composition and/or dynamics of EVs release. Furthermore, the identified glycosignatures of EVs could provide novel biomarkers for ovarian cancer.

## 1. Introduction

Extracellular vesicles (EVs) are produced by virtually all cells and a large body of evidence for their biological relevance has been obtained for immune, tumor or neural cells. The EVs may have different cellular origins, either from the multivesicular endosomes (designated as exosomes) or from budding off the plasma membrane (commonly known as microvesicles) [1]. Exosomes are in the range of approximately 30 to 150 nm whereas microvesicles have a more heterogeneous size distribution ranging from 50 to 2000 nm in diameter. Exosomes have been generally described to equilibrate at densities of 1.13 to 1.19 g/mL in sucrose gradients. In addition, EVs include apoptotic vesicles that usually have larger diameters, ranging from 50–5000 nm [1,2].

In view of the overlapping of several biochemical and biophysical characteristics, the discrimination of the different vesicle types is a particularly complex topic due to limitations of the techniques used for their purification. More recently, it has been suggested that also from endosomal origin there are different types of exosomes. Therefore, the methodology for isolation of pure vesicle fractions is a challenging topic and, currently, several techniques are used, including ultracentrifugation, ultrafiltration, gel exclusion chromatography, immunoaffinity isolation [3], and lectin affinity separation [4]. Because of the different methodologies used for EVs isolation and characterization, a consensus article aiming at standardization of sample collection, isolation, and analysis methods for EVs research has recently been published [5].

EVs present a specific protein, lipid and glycan composition. The content of EVs is the result of sorting mechanisms involved in their biogenesis and the cells from where they originate. Numerous studies on EVs composition of biomolecules have been made in recent years with accumulating results displayed in the database Vesiclepedia ([6]; http://www.microvesicles.org/) that contains data on protein, mRNA, miRNA, and lipid composition of extracellular vesicles (exosomes, ectosomes or shedding microvesicles and apoptotic bodies).The protein composition of EVs reflects in part the molecular mechanisms of biogenesis. In the formation of exosomes the membrane of endosomes invaginates with the production of intraluminal vesicles resulting in the appearance of multivesicular endosomes (reviewed in [7]). Those luminal vesicles designated as exosomes, have the same membrane topology as the plasma membrane, and will appear in the extracellular environment as the result from fusion of the multivesicular endosomes with the plasma membrane. The biogenesis of intraluminal vesicles requires the action of proteins from the endosomal sorting complex required for transport (ESCRT). ESCRTs consist of four protein complexes ESCRT-0, -I, -II, and -III and associated proteins [7,8]. ESCRT-0 binds ubiquitinated cargo proteins, PI(3)P and clathrin. It initiates cargo sorting by recruiting ESCRT-I by binding its protein component Tsg101. ESCRT-II binds ESCRT-I and ESCRT-III. Proteins from the ESCRT-III complex are recruited from the cytosol and polymerize thus forming filaments that induce vesicle formation [7]. Therefore, exosomes contain proteins involved in biogenesis (e.g., Alix and Tsg101), and also proteins involved in membrane fusion and transport (e.g., RabGTPases, annexins, flotillin). They also contain cytoplasmic proteins including cytoskeleton and heat-shock proteins (e.g., hsc70) [1]. Such proteins are often used as markers during EVs purification.

The budding mechanisms involved in the release of microvesicles from the plasma membrane are less well described at the molecular level. It is possible that they are related to virus budding mechanisms. Indeed many of the studies published on the characterization of exosomes, include most likely a mixture of vesicles from endosomal and plasma membrane origin.

Another class of proteins that is particularly enriched in EVs are tetraspanins that are membrane proteins with four transmembrane domains. Tetraspanins are palmitoylated and usually are glycosylated [9,10,11]. They associate with themselves and other proteins, including integrins, immunoglobulin-superfamily receptors, and metalloproteinases, and interact with cholesterol and gangliosides, thus forming specific membrane platforms [10]. Thus, tetraspanins, such as CD63, CD9, CD81, and CD82 have been widely used as EVs markers [12]. Evidence from the literature also suggested that tetraspanins are involved in EVs biogenesis [1]. For example, in the absence of the ESCRT machinery cells are still able to produce multivesicular endosomes and CD63 positive exosomes [13]. In addition, tetraspanins have been implicated in protein sorting into the exosomes more specifically selected CD81 ligands are depleted from exosomes in CD81-deficient cells [14].

EVs are generally enriched in sphingomyelin, cholesterol, phosphatidylserine, phosphatidylinositol, phosphatidic acid, saturated fatty acids, ganglioside GM3, ceramide, and in GPI-anchored proteins [15]. Furthermore, ceramide was shown to play an important role in exosome formation via a mechanism independent of the ESCRT machinery in a neural cell line [16].

EVs from different cell types have consistently been found to contain characteristic glycan signatures that are distinct from the parental cell membranes. Initial findings showed distinct prion protein glycoforms incorporated into exosomes [17]. Furthermore, it was found in T cells, melanoma, and colon cancer cells that, although the overall glycomics composition of EVs was related to the parental cells, there were distinct glycosignatures for EVs relative to the parental cell membranes, as evaluated by lectin array analysis. An enrichment of high mannose, polylactosamine, α2,6-linked sialic acid, and complex *N*-glycans was noted but also a depletion of terminal blood group A and B antigens [18,19]. In ovarian carcinoma cells specific sialoglycoproteins were found associated with EVs and specific glycosylation signatures were detected in EVs, relative to plasma membrane or microsomal glycoproteins [20,21].

Although EVs contain specific biomolecules as a result of biogenesis, they also share common structural features with their parental cells, which are distinct from those found in other cell types. Since EVs are found in extracellular compartments including body fluids, they could become very useful targets for biomarker identification. The emerging potential of EVs as biomarkers has been suggested in several diseases, most notably in cancer where increased amounts of EVs are produced by tumor cells [22,23,24].

In the present work we showed that EVs from ovarian carcinoma cells have specific glycosignatures using lectin blotting, which may constitute potential biomarkers. Furthermore, we observed that the glycosylation inhibitor kifunensin (KIF) had an impact in EVs composition.

## 2. Results

### 2.1. Production, Purification and Characterization of EVs

**Figure 1:**
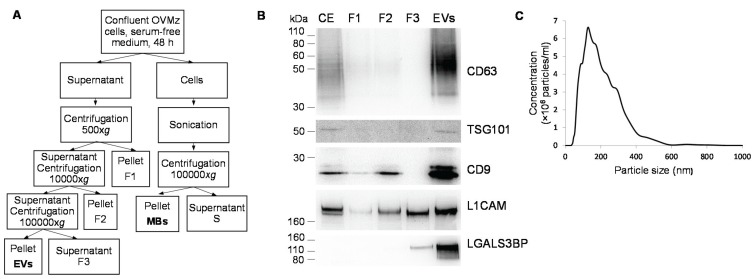
Isolation of EVs from OVMz cells. (**A**) Diagrammatic representation of the isolation procedure; (**B**) Immunoblotting of EVs markers in cellular extracts (CE), fractions collected during the purification (F1, F2, F3) and extracellular vesicles (EVs). Three µg of total protein were applied per lane with the exception of CE where ten µg of total protein were used. Detection was by the chemiluminescent method. Results were representative of two experiments; (**C**) NTA distribution profile of a representative population of EVs diluted in sterile PBS and analyzed using NanoSight NS500 equipment.

EVs were isolated from confluent monolayers of OVMz ovarian carcinoma cells grown in serum depleted medium for 48 h. The supernatant was centrifuged at 500× *g*, 10,000× *g*, and 100,000× *g* as previously described [21] (Figure 1A) and the different fractions were analyzed by immunoblotting with antibodies against EVs markers, CD63, Tsg101, CD9, and L1CAM. The results showed that EVs were strongly enriched in the 100,000 g pellet (Figure 1B). Furthermore, the sialoglycoprotein galectin-3-binding protein (LGALS3BP, encoded by the *LGALS3BP* gene, and also known as MAC2BP), which was previously identified as an EVs marker in ovarian carcinoma SKOV3 cells [21] was also found strongly enriched in EVs from OVMz cells (Figure 1B).

To characterize particle size distribution, the samples of EVs were measured by nanoparticle tracking analysis and a representative plot is shown in Figure 1C. The EVs exhibited a heterogeneous population in the range between 30 and 900 nm. The maximum of the major peak ranged between 91 and 191 nm with an average of 145 ± 26 nm (*n* = 24 plots from four EVs isolations). The heterogeneity can be related to the degree of sample purification, since the pellet of the 100,000× *g* centrifugation consisted of a crude mixture of different populations of vesicles with endosomal and plasma membrane origin that have been reported to have heterogeneous sizes [1,2].

**Figure 2:**
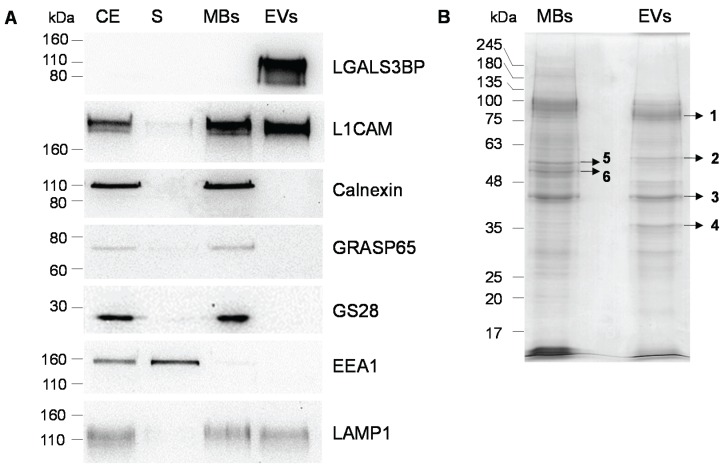
Comparison of protein profiles of MBs and EVs from OVMz cells. (**A**) Immunoblotting of cellular extracts (CE), post-100,000 g supernatant from MBs isolation (S), MBs and EVs. Ten µg of total protein were applied per lane with the exception of EVs in the incubation with LGALS3BP where three µg of total protein were used. Detection was by the chemiluminescent method. Results were representative of three experiments; (**B**) SDS-PAGE analysis of proteins of MBs and EVs. Ten µg of protein were applied per lane. Protein detection was with Coomassie R-250.

For comparison total cell membranes (MBs) were obtained from OVMz cells after sonication as described [25] (Figure 1A). To confirm the composition of the MBs immunoblotting with antibodies against markers for cellular membrane compartments were performed (Figure 2A). MBs were found to contain endoplasmic reticulum (detected by anti-calnexin), Golgi apparatus (anti-GRASP65 and GS28), lysosomes (LAMP1), and plasma membrane (L1CAM). For early endosomes only a faint band with anti-EEA1 was detected in the MBs, whereas it was found in the corresponding supernatant, probably as result of sonication since EEA1 is a peripheral protein. L1CAM and LAMP1 were also detected in EVs as previously described ([26], Vesiclepedia). LGALS3BP was only detected in the EVs but not in MBs. LGALS3BP is a protein from the cellular matrix that was found to interact with other proteins from the extracellular matrix, such as integrins. Since it does not contain transmembrane domains it would be expected not to be found in the MBs fraction. However, it is strongly enriched in EVs, probably via interaction with other proteins either from the extracellular matrix, such as collagens IV, V and VI, fibronectin [27], which have also been found in EVs (Vesiclepedia), or lectins, namely galectin-3, that have already been described in exosomes [25]. Since LGALS3BP was found soluble in the post-100,000 g supernatant (F3, Figure 1A) it possibly associates with the EVs extracellularly, which would explain no/low detection in the cell extracts and fractions of MBs isolation (Figure 2A).

The specific marker profiles confirmed the identity of the MBs and EVs fractions. These were further analyzed by SDS-PAGE and staining with Coomassie Blue R-250 and different profiles for the total proteins were found (Figure 2B). Major bands that were highly enriched in EVs or MBs were identified by MALDI-TOF/TOF analysis after trypsin digestion (Table 1, Table S1 and Table S2). Most proteins were from cytoplasmic origin with the exception of LGALS3BP, which is from the extracellular matrix, and their presence in EVs has already been described in Vesiclepedia.

**Table 1:** List of proteins identified in EVs and MBs from OVMz cells using MALDI-TOF/TOF after SDS-PAGE separation using MALDI-TOF/TOF. Bands were excised from the gel shown in Figure 2B.

Gel Band	Protein Name	UniProt Identifier	Gene Name	Nominal Mass (M_r_)	Protein Score	Sequence Coverage (%)	Queries Matched	Vesiclepedia
1	Galectin-3-binding protein	LG3BP_HUMAN	*LGALS3BP*	65,289	460	27	13	+
	Alpha-actinin-4	ACTN4_HUMAN	*ACTN4*	104,788	50	10	8	+
2	Pyruvate kinase PKM	KPYM_HUMAN	*PKM*	57,900	47	18	7	+
3	Actin, cytoplasmic 2	ACTG_HUMAN	*ACTG1*	41,766	447	50	15	+
	Actin, alpha cardiac muscle	ACTC_HUMAN	*ACTC1*	41,992	166	19	7	+
	Actin, alpha skeletal muscle	ACTS_HUMAN	*ACTA1*	42,024				+
4	Glyceraldehyde-3-phosphate dehydrogenase	G3P_HUMAN	*GAPDH*	36,030	157	52	16	+
	Ezrin	EZRI_HUMAN	*EZR*	69,370	69	3	3	+
	Moesin	MOES_HUMAN	*MSN*	67,778				+
	Radixin	RADI_HUMAN	*RDX*	68,521				+
5	Vimentin	VIME_HUMAN	*VIM*	53,619	415	48	21	+
6	Keratin, type II cytoskeletal 8	K2C8_HUMAN	*KRT8*	53,671	84	21	12	+
	Tubulin alpha-1B chain	TBA1B_HUMAN	*TUBA1B*	50,120	74	22	7	+
	Tubulin beta-4A chain	TBB4A_HUMAN	*TUBB4A*	49,554	54	18	6	+
	Tubulin beta-4B chain	TBB4B_HUMAN	*TUBB4B*	49,799				+

The presence of LGALS3BP in EVs from OVMz cells was in agreement with the immunoblot analysis (Figure 2A). Although the mass calculated from the amino acid sequence of the protein without the signal sequence is 63,277, it was detected at approximately 110 kDa by SDS-PAGE indicating that it was heavily glycosylated.

**Figure 3:**
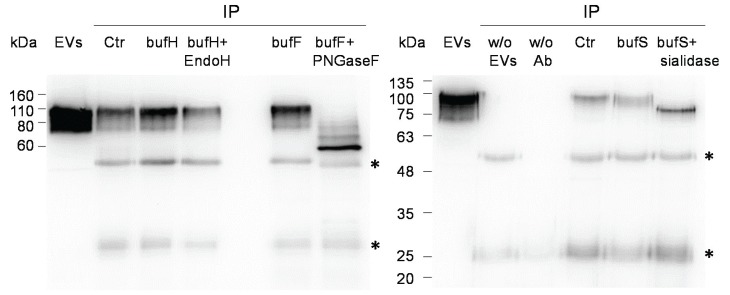
Deglycosylation of immunoprecipitated LGALS3BP. LGALS3BP was deglycosylated with Endo H, PNGase F, and sialidase from *V. cholerae* after immunoprecipitation from EVs (Ctr). The input EVs contained three µg of total protein. As control for the digestion the immunoprecipitate was incubated with the corresponding buffer (bufH for Endo H, bufF for PNGase F and bufS for sialidase). The controls of the immunoprecipitation without EVs (*w*/*o* EVs) and without antibody (*w*/*o* Ab) were also shown in the second panel. The blots are representative of two (Endo H) or four (PNGase F and sialidase) experiments. Immunoglobulin G bands are represented with *.

In order to investigate the type of glycosylation, LGALS3BP from EVs was immunoprecipitated and digested with endoglycosidase H (Endo H), peptide *N*-glycosidase F (PNGase F), and *Vibrio cholerae* sialidase. The protein was not sensitive to digestion with Endo H, showing the absence or very low amounts of high mannose glycans. Digestion with peptide *N*-glycosidase F caused a shift to a mass of approximately 60 kDa corresponding most likely to the fully deglycosylated form. Fainter bands at intermediary molecular masses were also detected, probably representing incomplete deglycosylation due to the large size of the protein. However, we cannot rule out other post-translational modifications. Digestion with *V. cholerae* sialidase also caused a downward shift of LGALS3BP indicating the presence of sialic acid (Figure 3B).

### 2.2. Glycosignatures of EVs and MBs

**Figure 4:**
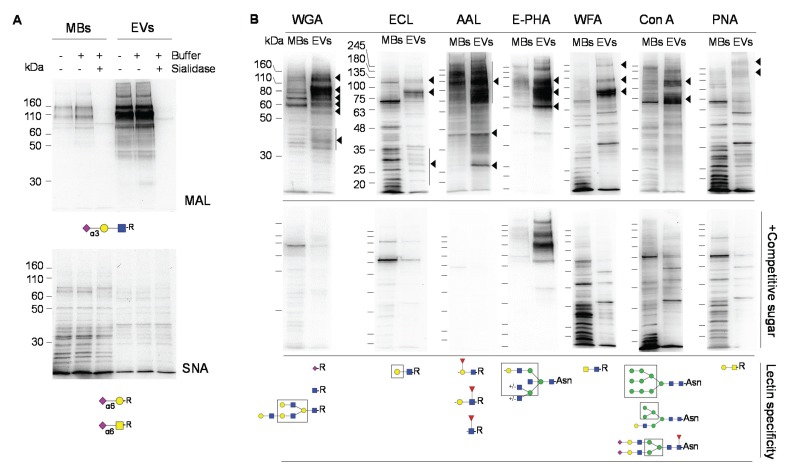
Comparison of glycosignatures from MBs and EVs. A. Lectin blotting with biotinylated MAL and SNA. As control for the lectin blotting the samples were desialylated with sialidase from *V. cholerae*. B. Lectin blotting with biotinylated WGA, ECL, AAL, E-PHA, WFA, and PNA, and non-biotinylated Con A (upper panels). Controls with competitive sugars as indicated in Material and Methods, are shown in the lower panels. Lectin specificities [28] are shown below the blots. Glycan representation is according to the nomenclature of the Consortium of Functional Glycomics. The lanes contained ten µg of protein. Detection was by the chemiluminescent method. Major specific bands are indicated on the right with arrowheads. The blots are representative of at least three experiments.

EVs and MBs were analyzed by lectin blotting with a panel of lectins to investigate EVs specific signatures. The lectin from *Maackia amurensis* (MAL) revealed the enrichment of several sialoglycoproteins with sialic acid in α2,3-linkage in EVs and a strong band was detected at approximately 110 kDa (Figure 4A), which probably consisted of LGALS3BP. On the contrary, the detection with *Sambucus nigra* agglutinin in EVs was faint and it was at the same level as the control after incubation with sialidase, which indicated the absence of sialic acid in α2,6-linkage in EVs from OVMz cells (Figure 4A). This result differed from ovarian carcinoma SKOV3 cells [21].

Wheat germ agglutinin (WGA), which binds sialic acid, also revealed a distinct profile between MBs and EVs, and a strong band appeared at approximately 110 kDa that is compatible with LGALS3BP (Figure 4B). The glycoprotein profiles with ECL, AAL, E-PHA, WFA, Con A, and PNA with glycan specificities shown in Figure 4 (below the blots) were also distinct between MBs and EVs fractions. Major bands that were enriched in EVs relatively to MBs and that decreased/disappeared in the presence of the competitive sugar are indicated on the right of the panels with arrowheads.

The presence of bisecting-GlcNAc, detected with E-PHA, was previously proposed as a potential marker for ovarian cancer cells [29,30], and also found in SKOV3 cells [21], was also detected here in OVMz cells and was strongly enriched in the EVs fraction (Figure 4B). Furthermore, the LacdiNAc structure detected with WFA previously found in SKOV3 cells [31], was also found here in EVs from OVMz cells (Figure 4B). Finally, PNA, which binds the T antigen, also detected broad bands above 180 kDa in EVs.

### 2.3. Effect of the Glycosylation Inhibitor Kifunensine

KIF is an *N*-glycosylation inhibitor that prevents the processing of *N*-glycans from high mannose to complex glycans, since it inhibits the α-mannosidase I enzyme from the endoplasmic reticulum. To investigate the impact of the type of *N*-glycosylation on (glyco)protein composition of exosomes we tested the effect of 5 µM KIF on the cell cultures. As control cell concentration, cell viability and total protein were estimated. The number of cells was found to be 6.1 × 10^5^ ± 0.5 × 10^5^ cells/well or 5.4 × 10^5^ ± 0.6 × 10^5^ cells/well in the absence or presence of KIF, respectively, but the difference was not significant using the unpaired t test (*n* = 6; *p* = 0.0662). Furthermore, KIF did not affect cell viability (99 ± 1%, *n* = 18 or 99 ± 1%, *n* = 6 in the absence or presence of KIF, respectively). The amount of protein in the EVs fraction was 48 ± 12 µg protein/T75 (*n* = 8) and 57 ± 3 µg protein/T75 (*n* = 6), in the absence or presence of KIF, but the difference was not statistically significant using the unpaired *t* test (*p* = 0.0928).

**Figure 5:**
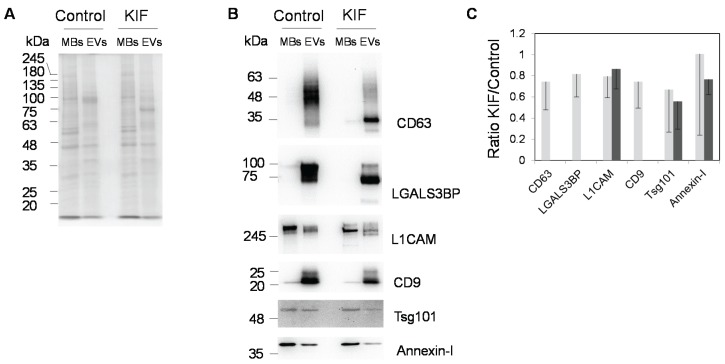
Effect of kifunensin on the protein profiles from MBs and EVs. (**A**) SDS-PAGE analysis. Protein staining was with Coomassie Blue-R250. Five µg of protein were applied per lane. KIF was used at 5 µM concentration; (**B**) Immunoblot analysis. Three µg of total protein were applied per lane; (**C**) Semi-quantitative analysis of the ratio between band intensities in the presence or absence of KIF using Image J software. Representative blots (**B**) average and standard deviation (**C**) from six experiments are presented. Light and dark grey corresponded to EVs and MBs, respectively.

Concerning the effect of KIF on the detection of several *N*-glycosylated (CD63, LGALS3BP, L1CAM and CD9) and non-*N*-glycosylated (annexin-I and Tsg101) EVs markers (Figure 5), clear differences were detected. First, the concentration of KIF used was found to efficiently inhibit *N*-glycosylation since CD63, LGALS3BP, and L1CAM had higher migration in SDS-PAGE in the presence of the inhibitor. The higher migration was due to lower molecular mass of high mannose glycans (e.g., Man_9_GlcNAc_2_ has 1883 Da), relative to complex glycans (e.g., complex sialylated diantennary with proximal fucose has 2369 Da or complex sialylated tetraantennary with proximal fucose has 3681 Da). Furthermore, although all proteins were still detected in the EVs in the presence of KIF several were detected at lower levels (Figure 5A). Semi-quantification of the bands from six replicates using Image J software 1.48v (Wayne Rasband, National Institutes of Health, Bethesda, MD, USA) revealed a trend towards a decrease of CD63, LGALS3BP, L1CAM, CD9, and Tsg101, whereas the difference for annexin-I was less evident (Figure 5B).

## 3. Discussion

### 3.1. EVs Glycosignatures and Glycoprotein Sorting Mechanisms

In the present article we have isolated and characterized EVs from ovarian carcinoma OVMz cells, grown in monolayer, with respect to their glycoprotein content and the type of glycosylation. We have found that EVs have specific glycosignatures compared to total membrane proteins using lectin blotting. Furthermore, we observed that inhibition of processing of high mannose to complex glycans caused changes in the dynamic composition of EVs.

Results from the literature had already reported specific glycan signatures for EVs from different cell lines, such as T-cells (Jurkat, SupT1 and H9), colon cancer (HCT-15 and HT-29), skin cancer (SkMel-5) [19], and ovarian carcinoma SKOV3 [21] cells. It is possible that the specific glycosignatures are the result of glycan-based sorting mechanisms that could involve cellular lectins, such as galectins. Galectin-3 and galectin-4 were detected in EVs [19] and galectin-5 has been proposed to mediate the concentration of poly-*N*-acetylactosamine containing glycoproteins into reticulocytes [32]. LGALS3BP, which binds galectin-3 and has previously been found abundantly expressed in EVs ([21,33], Vesiclepedia), was also found in the present work on OVMz cells. In agreement with this perspective lectins are involved in other sorting mechanisms within the cell. For example, several lectins including galectin-4, which is raft associated, and galectin-3, which is raft independent, are involved in apical targeting of glycoproteins in polarized cells [34]. More recently, sialylation of *N*-linked glycans was found to mediate apical targeting of endolyn via a galectin-9-dependent mechanism [35]. On the other hand, *N*-glycans were also shown to promote the trafficking of the urea transporter UT-A1 into membrane lipid raft subdomains [36]. Therefore, a pathway of glycoprotein sorting to EVs may be related to its glycan moiety as an alternative to the well characterized pathway based on the ESCRT machinery. The hypothesis would be that glycans interact with specific lectins, which promote the specific sorting of the carrier glycoproteins into exosomes or microvesicles at the endosome or at the plasma membrane, respectively. Whether that specific sorting would involve a previous enrichment into specific membrane domains (tetraspanin platforms or detergent resistant domains) could be a possibility. However, experimental evidence is still lacking at this point in support of these possible mechanisms.

The treatment of OVMz cells with kifunensine, which prevents the processing of high mannose to complex glycans, caused relatively decreased levels of the *N*-linked glycoproteins CD63, LGALS3BP, L1CAM, and also of the non-glycosylated exosome marker Tsg101 in the EVs, whereas the same effect on the non-glycosylated protein annexin-I was not observed. The results may be due to impaired sorting of specific glycoproteins into EVs. In agreement with this hypothesis, results from another group showed that the alpha-mannosidase I inhibitor, deoxymannojirimycin, caused decreased levels of the glycoprotein EWI-2 in EVs from Sk-Mel-5 cells, although its level on the cell surface did not appear to be affected [25]. However, these authors found LGALS3BP dramatically increased on EVs in the presence of the inhibitor much in contrast to our findings. This difference could be due to the different cell line used since non-treated OVMz cells have much higher levels of LGALS3BP than those reported for Sk-Mel-5 cells [25].

The decreased levels of specific glycosylated and non-glycosylated proteins that we observed in the presence of kifunensine could also result from changes in the composition of EVs into specific subpopulations of vesicles from endosomal (exosomes) or plasma membrane (microvesicles) origin or apoptotic vesicles. Since we applied the same amount of total protein per lane, the detected decrease of certain proteins could be due to reduced number of the vesicles where it is sorted, concomitant, or not, to the increased number of vesicles where it is not sorted. Further studies are required to explore this challenging topic, whether there is impaired sorting of certain glycoproteins into EVs caused by kifunensine, or if the cell dynamics are changing as a response to the inhibitor in a way that the composition of the pool of EVs is altered.

### 3.2. EVs Glycosignatures and Disease Diagnosis

EVs are carriers of potential disease biomarkers that include miRNA, proteins, glycoproteins [21,23,24,37,38], and protein glycosylation [21].

Here, we have detected the presence of bisecting-GlcNAc-containing glycans in EVs from OVMz cells. This type of structure has already been found by our group in EVs from ovarian carcinoma SKOV3 cells [21] and bisecting-GlcNAc-containing glycans. They were proposed as potential markers for ovarian cancer since tissues of serous and endometrioid ovarian carcinoma patients express this structure [29,30]. We also found the LacdiNAc structure in glycoproteins from OVMz EVs. This structure has been found in the *N*-glycans from tumor-associated glycoproteins including glycoproteins from ovarian carcinoma SKOV3 cells [31]. Furthermore, we detected the T antigen in OVMz EVs, and this carbohydrate structure is known to be increased in cancer [39].

Work by other groups has emphasized the importance of EV glycosylation as a disease biomarker. In this context, glycosylation of urine exosomes was studied by flow cytometry and lectin microarray and showed promising results for future studies on biomarkers for autosomal dominant polycystic kidney disease [40]. On the other hand, urinary microvesicles from patients with a deficiency in the galactose-1-phosphate uridyltransferase (GALT) gene showed dramatic shifts from prevalent high-mannose-type glycans found in healthy subjects towards complex-type *N*-linked glycosylation in a differential semiquantitative *N*-glycomics study of membrane proteins. These *N*-glycosylation shifts were not observed on the Tamm Horsfall glycoprotein, which showed predominant high-mannose-type glycosylation with M6 [41].

Concerning glycoproteins, exosomes derived from ovarian cancer patients carry the putative cancer marker glycosylated molecules CD24 and EpCAM, supporting their potential in diagnostics [42,43]. Here, we found that the sialoglycoprotein galectin-3-binding-protein was an abundant component of OVMz EVs as it was found in EVs from ovarian carcinoma SKOV3 cells [21]. High expression levels of galectin-3 binding protein are associated with a shorter survival, the occurrence of metastasis or a reduced response to chemotherapy in patients with different types of malignancy [44].

Potential biomarkers have been identified in EVs from several types of cancer, namely ovarian, colorectal, breast, pancreatic, and urogenital cancers including bladder and prostate cancer, glioblastoma, melanoma, lung adenocarcinoma, and esophageal squamous cell carcinoma [23,24,37,38,45]. There is also evidence that EVs isolated from the blood of glioblastoma patients can serve as a surrogate for primary tumor mutations and a predictive metric of treatment-induced changes [46].

EVs also provide potential biomarkers for other diseases other than cancer, such as neurological diseases, including ischemic stroke and multiple sclerosis [47], kidney-related diseases [24] or cardiovascular diseases [48], and neurodegenerative diseases where they carry misfolded pathogenic proteins [49,50,51,52,53] and deregulated microRNAs [24]. The studies concerning glycosylation of biomolecules from exosomes in neurodegeneration are scarce. In this context early studies showed that specific glycoforms of prion protein were associated with exosomes [17].

### 3.3. EVs Glycosignatures and EVs Uptake by Other Cells

Once EVs are released into the extracellular environment, they have the capability to interact with other cells and are, subsequently, internalized, thereby delivering their biomolecules into the selected target cells. In this way they can act as carriers of disease-associated biomolecules including nucleic acids as mRNA and miRNA [54], or pathogenic proteins, such as oncogenic receptor EGFRvIII in glioma [55]. In neurodegeneration, EVs are also partially responsible by the transmission of misfolded pathogenic forms of proteins, such as Abeta and tau associated with Alzheimer’s disease, alpha-synuclein associated with Parkinson’s disease, and superoxide dismutase associated with amyotrophic lateral sclerosis [49,56], among cells. On the other hand, beneficial roles of EVs from healthy cells have also been discussed in the context of cancer and neurodegeneration [57]. Most noteworthy, EVs from mesenchymal stem cells have been advanced as an alternative to the cells in several disease models [58].

The transmission of biomolecules between cells via EVs requires initial recognition steps between the EVs and the target cells with the participation of proteins, glycans, and lipids, followed by internalization of EVs by endocytic pathways or membrane fusion [59]. In the present work we report that EVs from OVMz cells have specific glycosignatures and it is possible that the glycans have a functional role in interaction and uptake by other cells. In support of this hypothesis are observations from several groups, including our own. For example, glycans from exosomes, more specifically α2,3-linked sialic acid-containing moieties from B cell exosomes, were shown to be recognized by sialoadhesin CD169 on macrophages in the marginal zone of the spleen and in the subcapsular sinus of the lymph node suggesting a potential role of CD169 in the immune response to exosomal antigen [60]. This is in line with our previous observations that desialylation of exosomes caused a trend towards an increase in exosome uptake by ovarian carcinoma SKOV3 cells [20]. Thus, it is possible that the presence of α2,3-linked sialic acid in several sialoglycoproteins from OVMz cells could play a relevant functional role in exosome uptake by other cells. Other sugars have been shown to play a relevant role in EV uptake; for example, heparan sulphate proteoglycans, which are known to mediate virus entry into cells, also participate in EVs uptake. More specifically, heparan sulfate proteoglycans of the syndecan and glypican type from recipient cells, but not from exosomes, participated in EVs uptake by glioblastoma cells, and the uptake was specifically dependent on the 2-O and *N*-sulfation groups [61]. Heparin was also shown to inhibit uptake of exosomes by bladder cancer cells [62]. In addition, in macrophages exosome uptake was inhibited by lactose possibly by interfering with a mechanism of recognition involving galectin-5 [32], whereas d-mannose and d-glucosamine inhibited exosome uptake by dendritic cells and a C-type lectin was at least in part required [63].

A body of evidence supports the relevance of tetraspanins, integrins, and IgSF molecules, which are generally glycosylated molecules, in EV-recipient cell interactions [59].

In view of their properties EVs have been used as nanocarriers for delivery of biomolecules of therapeutic value [64]. This is particularly valuable for diseases of the central nervous system since EVs seem to be capable of crossing the blood-brain-barrier [65]. In addition, AAV vectors associated with EVs were found to be more efficient in transduction of cells than conventionally purified AAV vectors [66]. Other studies showed that exosomes can efficiently deliver miRNA to epidermal growth factor receptor-expressing breast cancer cells [67]. Systemic exosomal siRNA delivery reduced α-synuclein aggregates in the brains of the transgenic mice model of Parkinson’s disease [68]. Finally, anti-inflammatory drugs encapsulated in exosomes and applied to the nasal region were used for the treatment of brain inflammatory diseases [69].

In view of EV specific glycosignatures and their potential implications in interactions with recipient cells one approach worth being explored to improve delivery would be through the modulation of EVs glycosylation. Further studies are required to investigate this topic.

## 4. Materials and Methods

### 4.1. Cell Culture

Human ovarian cancer OVMz cell line was grown in Dulbecco’s Modified Eagle Medium high glucose (Sigma, St. Louis, MO, USA), supplemented with 10% fetal bovine serum (Gibco, Grand Island, NY, USA), 100 units/mL penicillin and 0.1 mg/mL streptomycin (Gibco), at 37 °C, in 5% CO_2_.

### 4.2. Preparation of Cellular Extract, Extracellular Vesicles and Total Cell Membranes

Cellular extracts were obtained by solubilization of cells in 50 mM Tris-HCl pH 7.5 buffer, containing 5 mM ethylenediamine tetraacetic acid, 1% Triton X-100, 0.02% protease inhibitors cocktail, Complete (Roche Diagnostics GmbH, Manheim, Germany) for 30 min, followed by centrifugation at 10,000× *g*, 10 min, at 4 °C.

For EVs production, OVMz confluent cells were cultivated for 48 h in serum-free medium. The supernatant was collected and successively centrifuged at 500, 10,000, and 100,000× *g*, for 10, 20, and 120 min, respectively, at 4 °C. The pellet of the last centrifugations consisted of the EVs fraction.

For the separation of total cell membranes (MBs), confluent cells were incubated with 0.5 M ethylenediamine tetraacetic acid (EDTA) pH 8.0, for 10 min, collected with a cell scraper and centrifuged at 500× *g*, 5 min. Then, cells were sonicated on ice with three cycles of 5 s, at 70% power, Branson Digital Sonifier Models 250/450 and 2 min pause in between cycles for cooling. MBs were collected as the pellet of a 100,000× *g* centrifugation, for 1 h. The diagrammatic representation of the procedures is shown in Figure 1A. The recovery of protein was approximately 130 µg total protein/T175.

The glycosylation inhibitor KIF (Sigma) at 5 µM was added to confluent cells in serum-free medium, for 48 h, in 24-well-plates to determine cell concentration and viability, or in T75 flasks for EVs production. Cell viability was estimated by the trypan blue exclusion assay. Statistical analysis was done using GraphPad Prism 6 (GraphPad Software Inc., La Jolla, CA, USA).

Protein concentration was determined by the bicinchoninic acid method.

### 4.3. Immunoblotting and Lectin Blotting Analysis

Proteins were analysed by SDS-PAGE and transferred to polyvinyledene fluoride membranes that were blocked for 1 h with 5% defatted dry milk (Nestle Portugal S.A., Linda-A-Velha, Portugal) in phosphate-buffered saline (PBS) with 0.1% Tween-20 (PBST) or in Tris-buffered saline (TBS) with 0.1% Tween-20 (TBST). The following antibodies were used: mouse anti-L1CAM (L1-11A) monoclonal (1:1000), mouse anti-CD63 monoclonal (1:500) (Invitrogen, Camarillo, CA, USA), mouse anti-CD9 monoclonal (1:5000), goat anti-human LGALS3BP polyclonal (1:2000) (R&D, Minneapolis, MN, USA), goat anti-Tsg101 polyclonal (1:200), goat anti-GRASP65 polyclonal (1:500), goat anti-calnexin polyclonal (1:500) (Santa Cruz Biotechnology, Santa Cruz, CA, USA), mouse anti-annexin-I monoclonal (1:5000), mouse anti-human LAMP-1 monoclonal (1:500) (BD Biosciences Pharmingen, San Diego, CA, USA), mouse anti-EEA1 monoclonal (1:1000), mouse anti-GS28 monoclonal (1:250) (BD Transduction Lab, San Diego, CA, USA). Secondary antibodies were sheep anti-mouse IgG coupled to HRP (1:4000) (Amersham, GE Healthcare Europe GmbH, Carnaxide, Portugal) or rabbit anti-goat IgG coupled to HRP (1:20000) (Sigma). Washings were with TBST or PBST. For annexin-I the buffer used was TBS. Detection was performed with the Immobilon Western chemiluminescent HRP substrate (Millipore, Billerica, MA, USA). CD63, and CD9 were analyzed in non-reducing conditions. Semi-quantitative analysis was done with the Image J software version 1.48v.

For lectin blotting, blots were blocked with 3% BSA biotin free (Carl-Roth, Karlsruhe, Germany) in TBST for 1 h. The they were incubated with the following lectins in TBST for 1 h: 25 µg/mL concanavalin A (Con A) (Sigma), 0.5 µg/mL *Phaseolus vulgaris* erythroagglutinin (E-PHA), 1 µg/mL *Wisteria floribunda* (WFA; Vector Laboratories, Burlingame, CA, USA), 5 µg/mL *Maackia amurensis* lectin (MAL), 0.5 µg/mL *Sambucus nigra* agglutinin (SNA), 0.1 µg/mL wheat germ agglutinin (WGA), 0.5 µg/mL *Erythrina cristagalli* lectin (ECL), 1 µg/mL *Aleuria aurantia* lectin (AAL), 0.5 µg/mL peanut agglutinin (PNA) (Galab Techonologies, Geesthacht, Germany). For Con A, E-PHA, and ECL TBST contained 1 mM CaCl_2_ /1 mM MnCl_2_ or 1 mM CaCl_2_ /1 mM MgCl_2_ for PNA. The blots were then incubated with 0.1 μg/mL streptavidin-peroxidase (Sigma). Washings were performed with TBST with or without salts. Detection was performed with the Immobilon Western chemiluminescent HRP substrate (Millipore).

As control of MAL and SNA specificity MBs and EVs were incubated with sialidase from *Vibrio cholerae*, as previously described [20]. As control of WGA, AAL, E-PHA, WFA, Con A, and PNA specificities, incubations were done in the presence of competitive sugars, respectively, 0.5 M *N*-acetylglucosamine, 0.1 M fucose, 0.4 M and 0.1 M *N*-acetylgalactosamine, 0.1 M methyl-α-D-mannopyranoside and 0.3 M galactose, after a pre-incubation of 15 min of the lectin with the sugar.

Gels were stained with Coomassie Blue R-250 (Merck, Darmstadt, Germany) and destained with 25% methanol and 7% acetic acid.

### 4.4. Immunoprecipitation and Deglycosylation of LGALS3BP

For each immunoprecipitation, 20 μL aliquot of Protein A/G-agarose beads (Santa Cruz Biotechnologies) were incubated with 5 μL of goat anti-human LGALS3BP polyclonal antibody (R&D) for 20 min, at 4 °C, with constant rotation, in RIPA buffer (50 mM Tris-HCl pH 7.5, 150 mM NaCl, 0.1% SDS, 1% sodium deoxycholate, 1% Triton-X 100, 0.02% protease inhibitors cocktail, Complete, Roche Biodiagnostics GmbH). These beads were then incubated for 1 h, at 4 °C, with RIPA-solubilized EVs (150 µg), which had been pre-cleared with 20 μL of Protein A/G-agarose beads for 20 min. Washings were done with RIPA buffer. For deglycosylation, beads were incubated with 0.5% SDS, 1% β-mercaptoethanol and 0.02% protease inhibitors cocktail (Roche Biodiagnostics GmbH), at 99 °C. After cooling, the beads were incubated at 37 °C overnight, either with 5 mU Endo H (Roche Diagnostics GmbH) in 50 mM sodium citrate pH 5.5, or with 2.5 mU PNGase F (Prozyme, Hayward, CA, USA) in 1% Nonidet P-40 and 50 mM sodium phosphate pH 7.5, 10 mM EDTA. For sialidase digestion, beads were incubated overnight, at 37 °C, with 15 mU sialidase from *Vibrio cholerae* (Roche Diagnostics GmbH) in 50 mM sodium acetate pH 5.5 containing 4 mM CaCl_2_.

### 4.5. Nanoparticle Tracking Analysis (NTA)

Concentration and size distribution of EVs were measured using a NanoSight NS500 (NanoSight Ltd, Amesbury, UK). The samples were diluted in sterile PBS to get a particle concentration in the instrument linear range (10^8^–10^9^ particles/mL). All measurements were performed at 22 °C. Sample videos were analyzed with the Nanoparticle Tracking Analysis (NTA) 2.3 Analytical software (NanoSight Ltd., Wiltshire, UK)—Release version build 0025. Videos of 60 s were acquired and the average of ten measurements was considered as a representative result. Capture settings (shutter and gain) were adjusted manually. The mean size and standard deviation values of the major peak were calculated by taking into account all measurements.

### 4.6. MALDI-TOF/TOF Analysis and Protein Identification

Following protein separation by SDS-PAGE, protein gel bands of interest (stained with Coomassie Blue R-250) were excised from the gels for further MALDI-TOF/TOF analysis as previously described [70] with minor modifications. Briefly, gel pieces were destained with 50% (*v*/*v*) acetonitrile and digested overnight with trypsin (6.7 ng/μL) at 37 °C. Tryptic extracts were subsequently desalted and concentrated using homemade POROS R2 (Applied Biosystems, Warrington, UK) microcolumns. Peptides were eluted from the column using 5 mg/mL α-Cyano-4-hydroxycinnamic acid (LaserBio Labs, Sophia Antipolis, France) in 50% (*v*/*v*) acetonitrile with 5% (*v*/*v*) formic acid, and were applied directly to a MALDI plate. Data were acquired in positive reflector MS and MS/MS modes using a 4800 plus MALDI-TOF/TOF (Applied Biosystems, Foster City, CA, USA) mass spectrometer and the 4000 Series Explorer Software v.3.5.3 (Applied Biosystems, Foster City, CA, USA). External calibration was performed using the calibration standards (Pepmix1; Laser BioLabs). The fifty most intense precursor ions from the MS spectra were selected for MS/MS analysis. Data were analyzed using Protein Pilot Software v. 4.5 (ABSciex, Framingham, MA, USA) and the Mascot search engine (MOWSE algorithm). The search parameters used were: monoisotopic peptide mass values, maximum precursor mass tolerance (MS) of 50 ppm and a maximum fragment mass tolerance (MS/MS) of 0.3 Da; Carbamidomethyl (C), Deamidated (NQ), Gln- > pyro-Glu (*N*-term Q), and Oxidation (M) as variable modifications. A maximum of two missed cleavages was allowed. The searches were performed against SwissProt protein database (547,357 sequences; 194,874,700 residues) with taxonomic restriction to Homo sapiens (20,200 sequences). Only MS/MS data were considered for protein identification. All proteins identified have at least: one peptide fragmented with a significant individual ion score (score > 32, *p* < 0.05) and a bold red peptide match, in order to eliminate duplicate homologous proteins.

## 5. Conclusions

EVs from ovarian carcinoma cells have characteristic protein glycosylation signatures, thus suggesting specific sorting mechanisms of glycoproteins into EVs. Furthermore, the glycan structures identified in the EVs may constitute potential markers for ovarian cancer and constitute targets for investigation in other tumor cell lines and in human tissues.

## Supplementary Files

Supplementary File 1
